# Efficacy of Community Treatments for Schizophrenia and Other Psychotic Disorders: A Literature Review

**DOI:** 10.3389/fpsyt.2013.00116

**Published:** 2013-10-09

**Authors:** Julio Armijo, Emmanuel Méndez, Ricardo Morales, Sara Schilling, Ariel Castro, Rubén Alvarado, Graciela Rojas

**Affiliations:** ^1^Adult Psychiatry, José Horwitz Barak Psychiatric Hospital, Universidad de Santiago de Chile, Santiago, Chile; ^2^Medical School, Universidad de Chile, Santiago, Chile; ^3^School of Public Health, Universidad de Chile, Santiago, Chile

**Keywords:** schizophrenia, psychotic disorders, community health services, community mental health centers, psychosocial interventions

## Abstract

**Background:** In Chile, the clinical guidelines “for the treatment of people from first episode of schizophrenia” aim to support individuals with schizophrenia to live independently, establishment occupational goals, and gain an adequate quality of life and social interaction. This requires the implementation of a treatment model that integrates psychosocial and pharmacological dimensions. Community intervention strategies ensure the achievement of these goals.

**Objectives:** This study compiles and synthesizes available scientific evidence from the last 14 years on the effectiveness of community intervention strategies for schizophrenia and related psychotic disorders.

**Methodology:** An electronic search was carried out using PUBMED, LILACS, and Science Direct as databases.

**Criteria of inclusion:** (i) randomized clinical trials, (ii) Community-based interventions, (iii) diagnosis of schizophrenia or related psychotic disorder (section F2 of ICD-10). Exclusion Criteria: (i) treatments exclusively pharmacological, (ii) interventions carried out in inpatient settings, (iii) bipolar affective disorder or substance-induced psychosis (greater than 50% of sample).

**Results:** Sixty-six articles were reviewed. Community strategies for integrated treatment from the first outbreak of schizophrenia significantly reduced negative and psychotic symptoms, days of hospitalization, and comorbidity with substance abuse and improved global functioning and adherence to treatment. In other stages, there were improved outcomes in negative and positive symptoms and general psychopathology. Psychoeducation for patients and families reduced the levels of self-stigma and domestic abuse, as well as improved knowledge of the disease and treatment adherence. Training focused on cognitive, social, and labor skills has been shown to improve yields in social functioning and employment status.

**Conclusion:** Community-based intervention strategies are widely supported in the treatment of patients with schizophrenia.

## Introduction

In Chile, there have been great advances in national mental health policies, which have culminated in the establishment of guaranteed treatment for four mental disorders: schizophrenia, starting with the first episode; depression in individuals over 15; abuse and dependence on alcohol and drugs in individuals younger than 20; and bipolar disorder in individuals 15 years of age or older (1).

The clinical guidelines “for the treatment of people beginning in the first episode of schizophrenia,” published in 2009, aim to support patients with schizophrenia so that they may live independently, establish and pursue occupational goals, increase social interaction, and achieve a reasonable quality of life. It is essential to have a comprehensive treatment plan, which includes psychosocial and pharmacological dimensions and assures the continuation of these two dimensions throughout the treatment process. It is therefore recommended that the psychosocial interventions include adherence support, art therapy, cognitive-behavioral therapy (CBT), cognitive rehabilitation, counseling and therapeutic support, family interventions, psychodynamic and psychoanalytic therapy, psychoeducation, and social skills training, and that, in line with the new community model for mental health, these interventions be carried out within the community (2).

The objective of this study is to review the existing scientific evidence on the effectiveness of psychosocial community treatments for schizophrenia and related psychotic disorders (F2 spectra of the ICD-10, F20 schizophrenia, F22 delusional disorder, F23 brief psychotic disorders, F25 schizoaffective disorder, F29 unspecified, non-organic psychosis).

## Methodology

A qualitative review of literature from 1999 to 2012 was performed. Even though we did not carry out a systematic review, we used PICOS criteria (P, participants; I, interventions; C, comparisons; O, outcomes; S, study design) from the PRISMA guidelines, for selection of the studies (3).

Our investigative question was: what is the effectiveness of non-pharmacological community treatments for the management of schizophrenia and related psychotic disorders?

The studies were selected according to the following inclusion criteria:

– Participant population: patients with schizophrenia and disorders with psychotic features (delusional disorder, schizoaffective disorder, brief psychotic disorder, schizophreniform disorder, unspecified non-organic disorder).
– Type of intervention: community mental health services.
– Study design: prospective randomized controlled clinical trials (CCT).
– Outcomes: clinical (symptoms), user satisfaction, adherence to treatment, unmet needs, social functioning, occupational functioning, cognitive functioning, use of services, quality of life, hospitalizations, costs.
– Publication period: 1999–2012.
– Language: English/Spanish

The studies went through a second screening in accordance with exclusion criteria, which ensured that the reviewed articles complied with the conditions established in the investigative question. For this reason, studies that included the following were excluded:

– Participant population: bipolar affective disorder (greater than 50% of the sample), substance-induced psychosis (greater than 50% of the sample)
– Type of intervention: exclusively pharmacological or inpatient treatment. Studies that described the design of protocols, without preliminary results.

PubMed was the principal search database, and LILACS and Science Direct were used as alternatives. For PubMed, the MeSH key words used were: “schizophrenia and disorders with psychotic features” and “community mental health services.” The search was filtered for year of publication (01 January 1999 until 31 December 2012), type of article (Clinical Trial, Randomized Controlled Trial), and language (English, Spanish).

There were 113 article hits in the PubMed search, 33 in LILCAS, and 216 in Science Direct. After the abstracts were reviewed, according to the exclusion-inclusion criteria, and screened for duplicate hits, there were 66 articles remaining (59 from PubMed, 6 from LILACS, and 1 from Science Direct). The entire text of each of the 66 articles was reviewed.

The process of data extraction was carried out by three qualified readers, who used a spreadsheet with corresponding instructions to compile information according to the following variables: author, participants, age, intervention, total number of patients, follow-up, outcomes, randomization, blinding, intention-to-treat analysis, analysis of groups at baseline (and if they are comparable), results (with the corresponding statistical data).

## Results

Of the 66 reviewed articles, 17 were randomized clinical trials of interventions for patients with first-episode psychosis, and the remaining 49 evaluated interventions designed for patients in other stages of the illness. All of the studies included pharmacological interventions as part of the treatment, alongside the psychosocial community interventions, which were considered the independent variable.

### Characteristics of the studies

Sixty-five of the articles were CCT and only one corresponded to an observational study, which was included given its relevance to the objectives of the review. Of the 65 CCTs, 63 were randomized, and 43 described some type of blinding. The 65 CCTs carried out a comparison of the baseline characteristics of the study groups, and in 55 of the articles, the groups were completely similar at baseline; only 10 articles presented significant differences in some of the studied variables at baseline. Intention-to-treat analysis was used in only 32 studies. The loss of patients during follow-up varied between 5.3 and 53%. The total number of subjects that participated in the selected CCTs ranged from 24 to 708 patients.

Systematic reviews were not included, since the objective of this review was to gather information coming solely from original studies.

### Interventions for first-episode psychosis

Seventeen of the reviewed articles evaluated interventions for first-episode psychosis (Table S1 in Supplementary Material).

A series of five articles covered the OPUS trial in Denmark, a large randomized clinical trial of a 2-year program of modified assertive community treatment (ACT), multi-family group psychoeducation, and social skills training. In comparison to standard treatment (ST), the intervention significantly reduced psychotic symptomatology [Scale for the Assessment of Positive Symptoms (SAPS)], and negative symptomatology [Scale for the Assessment of Negative Symptoms (SANS)], and improved global functioning (GAF). In addition, OPUS reduced the number of hospitalized days by 22%, and adherence to comprehensive treatment was 8% greater, with more user satisfaction (4). However, the effects on symptomatology were not sustained at the 5-year follow-up (5). Additionally, OPUS reduced substance use in a population with dual-pathology (6), and in the subgroup of high risk of psychosis (schizotypal disorder), the percentage of transitioned to psychosis at 2 years was only 25% (vs. 48.3% in the ST group). Multivariable analysis showed a reduced risk for transition into psychosis (RR: 0.36) (7). There were no significant differences in quality of life (8).

OPUS intensive treatment (IT) was compared with hospital based rehabilitation (HBR) and ST, and both HBR and IT showed improvements in negative symptomatology at 1-year follow-up. Additionally, all three groups showed high levels of user satisfaction in terms of quality of life. The IT group presented fewer days/bed (IT: −139.1, ST: −53.6; HBR: reference), a greater number of patients living independently (IT: 68.8% vs. HBR: 40.0% vs. ST: 37.0%), and higher quality of life scores. There were no significant differences in the use of coercive measures (9).

“The Lambeth Early Onset Team” (London, England) tested a biopsychosocial intervention consisting of ACT along with evidence-based interventions according to the needs of each patient (CBT, family counseling, vocational strategies), carried out by a multi-professional community mental health team, for people presenting to mental health services for the first or second time with non-organic, non-affective psychosis. After adjusting for sex, number of previous psychotic episodes, and ethnicity, results showed a reduction in the average number of readmissions vs. ST (specialized care 0.4 vs. controls 0.8) and the lower dropout rate (OR: 0.28) (10). The intervention group had more reduced negative symptoms (PANSS) and increased GAF, although the PANSS results were not maintained after adjusting for race, sex, or number of episodes. The outcomes were also better in satisfaction (Verona Service Satisfaction Scale), quality of life (MANSA), and pharmacological adherence. In addition, the patients maintained educational or work activities for a longer period of time than the control group (6.9 vs. 4.2 months) (11). Patients receiving specialized care reported a greater average number of significant others in their social network (2.40 ± 1.20 vs. 1.71 ± 1.06), which linear regression analysis correlated with significant improvement in total PANNS and GAF (β = 2.95, SE = 1.04), showing the importance of social network in clinical improvement (12). Nevertheless, the intervention did not maintain results after 5-years follow-up in the re-hospitalization rates or in the average number of days/bed (13). There were also no significant differences in cost when compared to ST (14).

The early detection education program for general practitioner associated with the Lambeth Early Onset Crisis Assessment Team (LEOCAT) significantly increased the number of general doctors that referred patients directly to mental health services (86.1 vs. 65.7%), as well as reduced the delay before treatment initiation (5.9% of the patients evaluated by trained doctors delayed starting treatment more than 3 months vs. 27.3% of patients evaluated by untrained doctors, *p* < 0.05). However, the average duration of untreated psychosis (DUP) was not changed, as the intervention impacted only the final phases of the DUP (15).

Another group in London, the “Croydon Outreach and Assertive Support Team” (COAST), carried out an intervention consisting of treatment with atypical antipsychotics, cognitive-behavioral psychotherapy, a family intervention, and social and vocational assistance, delivered by a multi-professional team that was available 24 h a day, 7 days a week. Upon comparison with usual treatment (TAU: caseload = 35, without psychotherapy, including only medication monitoring and access to services), it was found that both groups improved the PANSS outcomes of positive symptomatology and general psychopathology, and in the regression analyses, the treatment-time interaction was not significant for any of the measures. It is not clear why COAST could not demonstrate significant improvements over TAU. One possibility is that patients were referred to the service and many of them were relatively stable, suggesting that the specialist input provided was not always necessary for this particular sample (16).

The Early Psychosis Prevention and Intervention Centre (EPPIC), in Melbourne, Australia, was created to provide thorough community treatment for patients, age 15–29, with first-episode psychosis. One of the interventions was cognitively oriented psychotherapy for early psychosis (COPE), consisting of weekly 40-min sessions, which progress through four phases (engagement, assessment, adaptation, secondary morbidity). After the 4-year follow-up, there was improvement in all the evaluated clinical variables (positive, negative, general, and depressive symptoms; quality of life; social functioning; reduction in readmissions) but without significant differences with respect to the control group (17). In the same context, a vocational intervention of individual placement and support (IPS) was evaluated. It consists of a vocational support method that offers protected work according to seven key principles: focus on competitive employment; treatment open to anyone with a mental illness; work search beginning at entry into program; program integrated into the mental health treatment team; job search based on patients’ preferences; time-unlimited support, continuing after employment is obtained, adapted to needs of patient; and counseling regarding the use of social benefits. The intervention group presented a greater number of employed patients (IPS = 13 vs. TAU = 2), number of hours worked per week (median IPS = 38 vs. TAU = 2.5), number of total jobs received (IPS = 23 vs. TAU = 3), and total earnings (mean IPS = U$4,449 vs. TAU = U$3,615). Additionally, the intervention group worked for a longer duration (median IPS = 5 weeks vs. TAU = 0) and showed a reduction by 55% in the number of people who required social welfare payments as their main source of income (18).

Early Psychosis Prevention and Intervention Centre also implemented cannabis and psychosis therapy (CAP), a cognitive-behavior program to reduce harm resulting from the consumption of marijuana. The intervention was carried out in 10 weekly sessions, 20–60 min each, over the course of 3 months. The program resulted in a reduction of cannabis use, with a median of 4 days of use in the 4 weeks leading up to the start of the study vs. 2 days at the end of treatment, and 1 day in the 4 weeks before the 6-month follow-up. These results, however, were also observed in the group that only received psychoeducation (19).

The Graduated Recovery Intervention Program (GRIP) is a thorough yet flexible module-based program with CBT, psychoeducation, and social skills training that aims to improve illness management and facilitate functional recovery after a first psychotic episode. The intervention program consists of 36 individual sessions with four modules: commitment and well-being management, substance use, persistent symptoms, and functional recovery. Although the majority of mixed model analyses were not statistically significant, examination of within-group changes and effect sizes suggests an advantage for GRIP over TAU in improving functional outcomes. GRIP participants showed improvement in social functioning (Role Functioning Scale = −2.96) and social competence (Multnomah Community Ability Scale-Social Competence subscale = −3.23) and tended to have a reduced PANSS general psychopathology subscale. Additionally, the TAU group had twice as many hospitalizations as the GRIP group (GRIP = 4 vs. TAU = 9) (20).

### Interventions for other stage of illness

The remaining 49 reviewed articles evaluated interventions for patients in others stages of schizophrenia (Table S2 in Supplementary Material).

#### Assertive community treatment

A total of nine randomized clinical studies evaluated the efficacy of ACT in patients with more advanced states of psychosis. The principal outcomes used in five of the studies were heterogeneous (severity of symptoms, GAF, hospital admissions, continual contact with community network, housing stability, self-management, user satisfaction) with an evaluation time ranging from 1 to 3 years. Of the remaining studies, two evaluated interventions specifically targeted to a forensic subpopulation, one evaluated a group with dual-pathology, and the final evaluated cost-effectiveness of an intervention.

Three studies evaluated symptom severity (PANNS, BPRS, CGI-S), finding significant improvements in favor of the intervention group (21–23), which also showed significant improvements in GAF (SOFAS) (21, 23), user satisfaction (CSQ) (21, 22), quality of life (Q-LES), and occupation (23). However, the results were mixed in terms of number of admissions and number of days of hospitalization. The study of Botha et al. (21) showing fewer readmissions and fewer days hospitalized, while the study of Bhugra et al. (22) did not detect significant differences. However, this was probably due to the fact that the Bhugra et al. intervention was directed at a culturally vulnerable subpopulation (black population), 10% of which was made up of bipolar patients.

The two remaining studies showed an increase in the number of monthly contacts between the patients and ACT team and a greater number of monthly home visits (24), as well as an improvement in substance abuse (SATS scale) and a great percentage of patients living independently (25).

In a similar vein, the intervention directed toward patients with psychosis and dual-pathology showed a lower probability of poor adherence over time (26).

The application of ACT for a forensic population – “Mental Health Treatment Court” (MHTC) – did not show significant differences in the reduction of criminal behavior but did show significant improvements in quality of life (Lehman QOL Life Satisfaction Scale), psychological distress (BASIS-32), and drug consumption (27). At the 2-year follow-up, F-ACT (forensic ACT) showed a reduced number of imprisonments, an increased number of contacts within the community health system, and fewer days hospitalized. Though it was more expensive to treat patients in the community, the costs associated with imprisonment were reduced per patient (28).

Finally, the evaluation of cost-effectiveness in the REACT study, which defined effectiveness as user satisfaction in relation to cost, showed a greater cost per worker in ACT, greater cost of hospital care in ACT, and greater monthly costs in ACT. The cost-effectiveness ratio was U$747 for each extra unit of satisfaction offered by ACT, with greater user satisfaction in ACT (29).

#### Intensive case management

Intensive case management (ICM) interventions optimize ACT, through the inclusion of client-focused case management, manual-based interventions, and comprehensive post-discharge treatment. We reviewed 10 studies that evaluated ICM interventions: 6 evaluated clinical outcomes, 2 evaluated the average number of days hospitalized, 1 evaluated a forensic population, and 1 evaluated cost-effectiveness.

The six interventions which evaluated clinical outcomes were found to increase user satisfaction, lessen caregiver burden (30, 31), improve psychiatric symptoms (CPS-50, BPRS) (32), reduce unmet needs (CAN) (32, 33), improve GAF (MFIS), reduce disability (DI, WHODAS) (31, 32), decrease caregiver stress (GCS) (32), and improve quality of life (Quality of Life Scale, QoL) (32), without significant differences in suicidal conduct (suicide attempt or completed suicide) (34) or violent behavior (35).

With respect to hospitalized days, the UK700 study found that a reduced caseload had no impact on the average number of days hospitalized over the 2-year period (36), and one study which evaluated impact of ICM upon “heavy users” of acute psychiatric inpatient beds (the 10% of patients that were most frequently hospitalized in a determined time period) found a fourfold increase in community contacts. However, there were no significant differences in any of the other clinical outcomes (37). The inclusion of more severe patient groups in these studies could explain the lack of significant difference, given that in the UK700 study, excluding a subpopulation of forensic patients reduced the number of hospitalized days significantly (ICM = 30 days vs. TAU = 60 days, *p* = 0.02) (36).

In a forensics population, intensified services for court-ordered outpatient care did not show significant differences in the studied variables (arrests, quality of life, and symptomatology) in either group, and the percentage with re-hospitalizations was similar. These results, however, were influenced by the study limitation that no control measures were put in-place to assure the adherence of typically non-adherent patients, who generally ended up being re-hospitalized (38); a similar study found that patients who were more adherent to the outpatient care program had fewer readmissions (57% fewer readmissions), fewer days hospitalized (20 fewer days on average), and less probability of violent behavior (27 vs. 42%) or being victimized (24 vs. 42%) (39).

An evaluation of the costs associated with ICM in Hong Kong, China found that there were significantly greater average costs for the intervention group, when compared to the control group (40). Harrison et al. also showed that total costs were greater in the experimental group, due to increased spending on outpatient community and primary care services, and a greater number of visits to general practitioners in the experimental group (37).

#### Community re-entry module

Three articles evaluated the Community re-entry module (CRM), which consists of nurse-led psychoeducation sessions covering self-administration of medications, early signs of relapse, emergency relapse plans, how to find housing, continuing care in the community, stress reduction, and promotion of coping strategies upon discharge. In one study, CRM vs. standard rehabilitation showed improvements in knowledge and skills in medication and symptom management, as well as a significant improvement in the REHAB scale at 1-year follow-up, but there were no significant differences in symptomatology (41). In another study, CRM vs. standard group psychoeducation reported significant differences at the 2-year follow-up in symptomatology (PANSS), reemployment rate, insight, and social functioning. Additionally, there were lower rates of re-hospitalization and relapses (42). CRM vs. supportive counseling found improvements in all subscales of PANSS and social functioning in favor of the intervention group (43). The differences in symptomatology improvement are probably due to the fact that in one study, CRM was compared with an inpatient integrated rehabilitation program, which included arts and crafts, reality orientation therapy groups, and work assignments, while the other two studies compared CRM to interventions that were limited to one aspect of illness management. An advantage of CRM is that it is community-based, and all three studies showed improvements in social functioning.

#### Cognitive-behavioral therapy

Six studies evaluated the efficacy of CBT in patients with schizophrenia. The outcomes were mainly clinical: psychotic symptoms (PANSS), depression (Beck and Calgary Depression Scale for Schizophrenia), and anxiety (Social Interaction Anxiety Scale, Brief Social Phobia Scale, Brief Fear of Negative Evaluation Scale), relapses, psychosocial functioning, insight, and quality of life were also evaluated. Follow-up period varied between 1 and 3 years. Significant differences were found in positive symptomatology (44, 45), negative symptomatology (45–47), and depressive symptoms and suicide (45, 47); however, the reduction of depression symptoms was the only result that was still significant at the 18-month follow-up (45). CBT also showed better outcomes in global and social functioning (44, 45), a reduced number of hospitalizations (44, 46, 47), and improvement in insight about the disease and symptoms (45–47). Furthermore one study evaluated group CBT and found that it significantly improved anxiety, depression, symptomatology, and quality of life (48). There were no significant differences in the intervention costs or occupational recovery (45, 46).

Another study which evaluated a needs-based family cognitive-behavior intervention for caregivers showed a lower relapse rate (37 vs. 72%, NNT = 3), significant improvements in PANSS for both groups (*p* = 0.005), and better GAF in the intervention group, though without differences in the caregiver variables (49).

#### Cognitive rehabilitation and social cognition

Five studies evaluated the effects of interventions focused on cognitive rehabilitation and improving social cognition. The first study was non-randomized and investigated the influence of both factors in psychosocial rehabilitation programs. The rate of rehabilitative change was statistically significant at follow-up and high neurocognition and social cognition scores at baseline predicted higher rates of rehabilitative change. Similarly, more days of treatment were associated with higher rates of functional change (50).

The four remaining studies were randomized, with a 3- to 12-month follow-up, and evaluated three interventions: Social Cognitive Enhancement Training (SECT), Feuerstein’s Dynamic Cognitive intervention, and Cognitive Adaptation Training (CAT). SCET vs. standard rehabilitation showed better results in the Social Behavior Sequencing Task, which measures ability to use social sequential information, and in the Picture Arrangement Task (*p* < 0.05), which measures perceptual and sequential organizational ability (distinguishing what is essential from peripheral in social context) (51). Feuerstein’s Dynamic Cognitive Intervention showed significant differences in memory and thought processes [Learning Potential Assessment Device (LPAD) and the Raven Progressive Matrices and General Aptitude Test Battery (GATB)] and gains in employment (31 vs. 14%) and independent living (44.4 vs. 17%) (52). CAT includes interventions carried out for 14 days, over the course of 6 months, in the patients’ homes according to patient profile: apathy, disinhibition, and dysexecutive syndrome, measured by the Frontal System Behavior Scale and Wisconsin Card Sorting Test. CAT vs. ACT alone showed significant improvements in the secondary outcomes for both groups, in terms of needs (CAN), symptomatology (PANSS), and quality of life (Lehman Quality of Life Interview), but there were no significant differences between the groups in any outcome, including GAF (primary outcome) (53). A study comparing CAT vs. Global Environmental Support – GES (generic support, without individualized differences) and CAT vs. TAU showed that CAT and GES had higher average social and occupational functioning (SOFAS) with significant differences in comparison to ST, but without significant differences between the two intervention groups (54).

#### Psychoeducation regarding Illness

Five studies evaluated psychoeducational interventions: three investigated patient and family joint pscyhoeducation sessions, and two evaluated patient-only psychoeducation. The patient and family psychoeducation interventions had a follow-up between 9 and 24 months.

A training program designed for Latino patients and their families showed significant differences when compared to the control group, at the 9-month follow-up, in terms of symptomatology, symptom management, medication management, functioning level, and rates of re-hospitalization (5.1 vs. 22.2%) (55). There were, however, no significant differences in quality of life or caregiver burden between the intervention and control groups. In another study, multi-family group psychoeducation produced a lower rate of psychiatric hospitalization in comparison to ST, although the results were not statistically significant (56). Family psychoeducation program in six rural towns in China yielded greater adherence to treatment, less family neglect and abuse, and greater understanding of the mental illness (57).

The patient-only psychoeducation interventions – the Evidence-Based Practices Implementation Project in Japan (58) and a psychoeducation program for Korean Americans (59) – showed improvements in the intervention groups symptomatology (BPRS) (58, 59), GAF, quality of life (SF-36), quality of social relationships (LSS), and self-sufficiency in daily living activities (SECL) (58). The intervention groups also showed lower Stigma-Devaluation Scale scores and greater scores on the Family Crisis Oriented Personal Evaluation Scales (59).

#### Family therapy

We found only one study that evaluated the effectiveness of a yearlong systemic family therapy for schizophrenia. At the first year of follow-up, the users in the family therapy group had lower rates of relapse than the usual treatment group (15 vs. 65%, *p* = 0.03), fewer consultations with the psychiatric emergency department (5 vs. 40%, *p* = 0.02), and were more adherent to medication (100 vs. 65%, *p* = 0.009). These differences, however, were not maintained at the second year of follow-up (60).

#### Crisis intervention plans

Two studies evaluated the effectiveness of Crisis Intervention Plans.

Critical Time Intervention (CTI) is a time-limited intervention that aims to assure the continuity of users’ care, during the transition from institutional settings to the community. Technical “CTI Workers” are paired with patients before discharge and help the patients strengthen ties with formal and informal supports in the community. CTI was shown to significantly reduce the number of “homeless” nights in a prior publication by Susser et al. (71). CTI also improves negative symptoms (PANSS negative symptom subscale), compared to usual treatment (CTI = −2.6 vs. USO + 1, *p* = 0.02). However, when only the results of patients with a diagnosis of schizophrenia were analyzed, there were no significant differences (CTI = −2.9 vs. USO = +5, *p* = 0.70) (61).

Flood et al. evaluated the cost-effectiveness of joint crisis plans, which are formulated by the patient, caregiver, psychiatrist, and project worker. The results showed that the use of joint crisis plans is associated with fewer forced hospitalizations (13 vs. 27%, RR = 0.48) and fewer total hospitalizations (30 vs. 44%; RR = 0.69), though the latter was not statistically significant. The total cost of the 15-month follow-up was £7,264 (€10,616, U$13,560) for each participant in the experimental group, vs. £8,359 (€12,217, U$15,609) in the control group. The cost-effective analysis reveals that there is a 78% probability that these crisis planning programs are more cost-effective than the standard presentation of information, due to their role in reducing the probability of hospital admissions (62).

#### Computer-mediated community interventions

Four of the reviewed studies investigated the effectiveness of computer-mediated community interventions. One study evaluated an online support platform, and the others evaluated the DIALOG program, a computer-mediated intervention with structured communication between health workers and patients that also measures the patients’ satisfaction.

Griswold et al. assessed the use of an online support platform in an ACT program to connect patients to primary care services after a crisis episode. The support platform was staffed by caregivers, who were trained in interviewing, case evaluations, and managing patients in psychiatric emergencies, and provide information about care centers, facilitate access to primary care, perform psychoeducation, give information about diagnoses and treatment, follow-up (including a home visit), and connect patients with peers via community mental health and social services web sites. At the 1-year follow-up, the intervention group was more connected to support services than the control group (62.4 vs. 37.6%) (63).

The multi-center DIALOG study found that, a year after the computer-mediated intervention, patients with predominately negative symptoms had a greatly improved quality of life (MANSA), and that this result was primarily moderated by the quality of the therapeutic alliance at the beginning of treatment and by the duration of the illness. Additionally, patients who were most symptomatic at the beginning of treatment were found to have a reduced number of unmet needs (CANSAS), which was moderated by the presence of competitive employment and shorter illness duration (64). The study also reported improvements in the quality of life scales (65, 66) and user satisfaction (66).

#### Therapy to encourage adherence

Three studies evaluated the effectiveness of Therapy to Encourage Adherence. One study of adherence therapy did not find significant differences when compared with the control group, which only received health education in terms of quality of life, adherence, or psychopathology (67). A training program for community nurses in the medication management for schizophrenia patients vs. usual management training reduced patient symptomatology (PANSS) (68). A pharmacy-based intervention, which used a plan to help patients remember their prescriptions and medication schedule through calendar-based containers, was found to significantly increase the medication possession ratio (MPR) – the ratio between the number of days medicine was given to the patient and the number of days he or she should have received medication (69).

## Discussion

The literature review identified articles on a wide range of community psychosocial treatments for schizophrenia. While the studies were of diverse methodological quality, the majority complied with the standards of randomization, blinding, and initial group analysis, even though only half of the studies performed intention-to-treat analysis.

The main methodological limitation identified is the diversity of interventions and control groups used in each of the studies. In many investigations, the ST already involved community-based interventions, and the intervention group optimized various factors of the preexisting interventions. In the case of interventions for first-episode psychosis (OPUS, LEO, COAST, EPPIC, CAP, and GRIP), the “standard care” received by the control group was often also delivered by community mental health teams and had the following characteristics: increased caseload size of 20 to 35, no additional training in the management of early psychosis, and no specialized psychological, social, or psychoeducational interventions (such as COPE, IPS, CAP, and GRIP). For interventions targeting other stages of the disease, control groups were more heterogeneous, but shared some common general characteristics. In ACT and ICM, ST was also implemented by community mental health teams but differs from intervention groups in the following: greater caseloads, not home based, did not include assertive engagement or after-hours services, and finally, the frequency of contact with patients varied between every month to every 3 months, and depended on caseloads, rather than on patients’ needs. The control groups for CBT, CRM, cognitive rehabilitation, psychoeducation, family therapy, computer-mediated community interventions, and crisis intervention plans lacked for the specific intervention.

A second methodological limitation was the heterogeneity of sample sizes. On interventions for first-episode schizophrenia, major studies (OPUS, *N* = 547; LEO, *N* = 466) have sufficient number of patients to test the power of their results, but studies with smaller sample sizes (COAST, *N* = 59; COPE, *N* = 91; IPS, *N* = 41; CAP, *N* = 47; GRIP, *N* = 46) require additional investigations to support their results. In the case of interventions for other disease states, sample size variability is even greater. For ICM and ACT, while the studies of Botha et al. (*N* = 60), Bhugra et al. (*N* = 83), and Chan et al. (*N* = 62) had fewer than 100 subjects, most other studies – i.e., Lambert et al. (*N* = 120), Salyers et al. (*N* = 324), Walsh et al. (*N* = 708), etc. – had a larger sample size, giving to their results more weight. In terms of other types of interventions, the sample sizes of Malik et al. (*N* = 257) and Turkington et al. (*N* = 422) for CBT, Velligan et al. (*N* = 120) for CAT, and Dyck et al. (*N* = 106), and Mao Ran et al. (*N* = 357) for psychoeducation were noteworthy. The sample sizes for computed-mediated community interventions (Griswold et al. *N* = 151; DIALOG, *N* = 507), crisis plans interventions (Herman et al. *N* = 99; Flood et al. *N* = 160), and therapy to encourage adherence (Gray et al. *N* = 409; Valenstein et al. *N* = 118) were also quite significant.

Another limitation to consider in our study is the possibility of the existence of publication bias: the tendency to submit articles based on the direction or magnitude of the results, thus over-representing certain characteristics of the studies. The main potential risk factors for publication bias are (1) the characteristics of study design (sample size, type of control group, number of collaborating centers), (2) the characteristics of the researcher, (3) the financing source, and (4) strength of the results.

Firstly, to reduce study design bias, we attempted to perform a varied and exhaustive search to ensure the inclusion of studies with smaller samples, given the potential tendency to publish studies with larger sample sizes because its larger effect size. We also did not reject studies based on the characteristics of the control group and included both: multicentric and local studies. In terms of study design, the selected articles were only randomized clinical trials. The large variability of sample sizes and control groups, and the presence of local and multi-center studies, evidences the rigor with which we followed the search criteria to avoid publication bias. Second, we included young and senior researchers, from both the hospital and the community setting. Third, in terms of financing, the study did not receive direct funding; instead, it was financially supported by a regional mental health research network known as Rede Americas, which aims to support the careers and training of young investigators in Latin Americas. Finally, regarding to strength of the results, we have tried to include both results with statistical significance as well as those with non-significant or negative results, in order to avoid publication bias.

Unfortunately, it is not possible to calculate “fail-safe *N*” in our study, since that requires meta-analysis. We suggest in future researches carry out a meta-analysis of community treatments for schizophrenia, in order to quantify the results of our review.

Taking into account the aforementioned methodological limitations, it is important to assess of the applicability of the reviewed interventions in Chile. Our clinical guidelines recommend psychosocial community interventions for the recovery phase of schizophrenia, but the implementation of such interventions requires specialized human resources (such as case managers and cognitive-behavioral therapists). Our challenge, is training primary care professional and technical staff, to carry out the different interventions. Moreover, the inclusion of “peers” – individuals who also suffer from a severe mental illness – as rehabilitation monitors, also appears to be a alternative to improve human resources, introduce users into the community mental health service network, and encourage recovery (70).

Finally, it must be noted that most of interventions were carried out in the recovery and/or social insertion phase of schizophrenia treatment, but also in the acute phase, that did not exclude hospitalization as a therapeutic alternative. Interventions analyzed in this review and hospitalized care should not be viewed as mutually exclusive. Community-based treatment should be viewed as a way to enhance treatment of schizophrenia, primarily in the phases of recovery and social integration. We maintain that the analyzed studies are innovative and provide good quality scientific evidence.

## Conclusion

Community psychosocial interventions for schizophrenia have been found to be effective in reducing positive and negative symptomatology and general psychopathology, both after the first psychotic episode and in other phases of the illness. The effects on negative symptoms are of particular interest, since these community interventions can serve as a complementary alternative to manage refractory symptoms, which at times are unresponsive even to treatment with atypical antipsychotics.

Additionally, evidence shows that community-based psychosocial interventions significantly reduce relapses and hospital readmissions, increase contacts with the community mental health teams, and improve adherence to pharmacological treatment. They do not, however, reduce costs.

The majority of the evaluated interventions reported improvements in GAF, social functioning, and user satisfaction. Vocational interventions result in successful job reintegration, and psychoeducation interventions have been shown to increase understanding of the illness and crisis management. The results also indicate that incorporating a culturally accepted program for a subpopulation of family members of a patient with severe mental illness yields better results than usual treatment. Furthermore, devising crisis plans in conjunction with the patients themselves is an effective strategy to prevent and manage situations that often lead to decompensation, and in the case of acute episodes, to address them in a timely manner, thus improving symptomatic results and reducing the number of hospitalizations.

Cognitive-behavioral therapy yields excellent results in terms of improving negative and positive symptomatology, general psychopathology, and depressive and anxiety symptoms, and it has been found to reduce the rates of relapse and re-hospitalization. Group versions of CBT have similarly been shown to reduce anxiety and social anxiety, and systemic family therapy also has produced positive results.

Family interventions are also effective in improving patient adherence and in reducing relapses and the use of emergency services, highlighting the necessity of including family members, whenever possible, in all comprehensive treatment programs for schizophrenia.

Cognitive rehabilitation and social cognition-focused interventions, which have been found to restore memory processes and executive functions and to improve symptomatology and psychosocial outcomes, should be more widely investigated and implemented.

Further, the implementation of computer technologies to connect with patients with schizophrenia has reported good results, leading to increased contact with caregivers and improved quality of life and user satisfaction.

The reviewed community-based interventions, which integrate evidence-based treatment strategies, offer a comprehensive treatment approach for schizophrenia and have reported high levels of social and clinical recovery, thus making possible the therapeutic outcomes desired by individuals with schizophrenia and their families: the ability to live independently, establish and pursue occupational goals, establish social relationships, and improve quality of life.

## Conflict of Interest Statement

The authors declare that the research was conducted in the absence of any commercial or financial relationships that could be construed as a potential conflict of interest.

## Supplementary Material

The Supplementary Material for this article can be found online at http://www.frontiersin.org/Schizophrenia/10.3389/fpsyt.2013.00116/abstract
